# Species interactions differ in their genetic robustness

**DOI:** 10.3389/fmicb.2015.00271

**Published:** 2015-04-14

**Authors:** Lon M. Chubiz, Brian R. Granger, Daniel Segrè, William R. Harcombe

**Affiliations:** ^1^Department of Biology, University of Missouri – St. LouisSt. Louis, MO, USA; ^2^Bioinformatics Program, Boston UniversityBoston, MA, USA; ^3^Department of Ecology, Evolution, and Behavior, University of MinnesotaSt. Paul, MN, USA; ^4^BioTechnology Institute, University of MinnesotaSt. Paul, MN, USA

**Keywords:** cooperation, competition, genetic robustness, *E. coli*, *Salmonella*, community stability, metabolic modeling

## Abstract

Conflict and cooperation between bacterial species drive the composition and function of microbial communities. Stability of these emergent properties will be influenced by the degree to which species' interactions are robust to genetic perturbations. We use genome-scale metabolic modeling to computationally analyze the impact of genetic changes when *Escherichia coli* and *Salmonella enterica* compete, or cooperate. We systematically knocked out *in silico* each reaction in the metabolic network of *E. coli* to construct all 2583 mutant stoichiometric models. Then, using a recently developed multi-scale computational framework, we simulated the growth of each mutant *E. coli* in the presence of *S. enterica*. The type of interaction between species was set by modulating the initial metabolites present in the environment. We found that the community was most robust to genetic perturbations when the organisms were cooperating. Species ratios were more stable in the cooperative community, and community biomass had equal variance in the two contexts. Additionally, the number of mutations that have a substantial effect is lower when the species cooperate than when they are competing. In contrast, when mutations were added to the *S. enterica* network the system was more robust when the bacteria were competing. These results highlight the utility of connecting metabolic mechanisms and studies of ecological stability. Cooperation and conflict alter the connection between genetic changes and properties that emerge at higher levels of biological organization.

## Introduction

Microbes often lead highly social lifestyles, engaging in interactions that span the gamut from cooperation to conflict (Mitri and Foster, 2013). These interactions determine the composition and function of microbial systems, and influence communities that are critical for both natural and applied processes. Microbes primarily interact through the compounds that cells remove from and excrete into the environment (Klitgord and Segrè, 2011). As such, the dynamics of species interactions are intimately connected to the behavior of physiological networks inside of cells. However, it is unclear how sensitive species interactions are to intracellular perturbations and vice versa. Specifically, how robust to mutation are emergent community properties when species are competing vs. when they are cooperating? And conversely, how do species interactions influence the physiological robustness of species?

There has been a great deal of work on both the robustness of genomes and the stability of communities, but little work connecting the two (Klitgord and Segrè, 2010). Genetic literature typically focuses on the likelihood that mutations will alter a phenotype. Indeed, connecting phenotypes to their underlying genotypes is a challenge that has spanned generations of scientists (Mather, 1941; Ho and Zhang, 2014), and has gained new momentum now that sequencing has enabled genome-wide association studies (Evangelou and Ioannidis, 2013). Another systems level approach that has proven highly useful is genome-scale libraries of gene knockout mutants (Winzeler et al., 1999; Baba et al., 2006). These libraries have allowed analysis of questions from the number of essential genes (Baba et al., 2006), to the average number of genes that affect phenotypes of interest (Ho and Zhang, 2014). While it is largely appreciated that environment mediates the connection of genotype to phenotype, there has been little systematic study of genetic perturbations in a community context (Klitgord and Segrè, 2010). Does the robustness of a genome change when it is involved in different ecological interactions? Do genetic perturbations have similar effects in different social contexts? Studying genomic robustness in a community context may provide further insight into the forces shaping genomic architecture.

Ecology literature focuses on the stability of communities to environmental perturbations. The relative stability of communities depends on the metric of interest. It is commonly held that competitive interactions should stabilize total community biomass through “compensatory dynamics” (Gonzalez and Loreau, 2009). If one species reduces in density, a competitor will increase in density to fill the available niche space (but see Loreau and de Mazancourt, 2013). In contrast, community composition is thought to be more stable in obligate mutualisms (Shou et al., 2007; Harcombe et al., 2014). If species are dependent on each other for essential metabolites this can create frequency dependent dynamics that push the system toward a stable equilibrium ratio of species. However, Kim et al. used a tri-partite system to demonstrate that uncoupled rates of consumption and production can lead to highly unstable dynamics (Kim et al., 2008). These approaches tend to ignore the genetic mechanisms underlying the interactions (Klitgord and Segrè, 2010). Do mutations frequently change the nature of ecological interactions? Are cooperative or competitive systems more robust to genetic perturbations? Studying the genetic basis of community stability can provide insight into the molecular mechanisms that determine community composition and function, and may improve prediction of community dynamics.

Genome-scale models of metabolism offer an opportunity to connect physiological mechanisms to the emergent behavior of species interactions. A stoichiometric, metabolic network can be used to define feasible patterns of steady state metabolite flow through a population of cells and optimization calculations can then identify the physiological fluxes that maximize the biomass production capacity or that satisfy other optimality criteria (Orth et al., 2010). This technique of flux balance modeling has had noted success at predicting the effect of environmental and genetic perturbations on cell growth (Segrè et al., 2002; Joyce et al., 2006; Orth et al., 2010). Harcombe et al. recently developed an extension of flux balance modeling that can simulate inter-species interactions as emergent properties of the uptake, excretion and environmental diffusion of metabolites (Harcombe et al., 2014). This flexible platform, called COMETS (computation of microbial ecosystems in time and space), accurately predicted the experimentally measured equilibrium ratio of 2 and 3-species microbial consortia. The impact of mutation on species interactions can now be tested by simulating growth of a mutant model in the presence of a model of another bacterial species.

**Figure 1:**
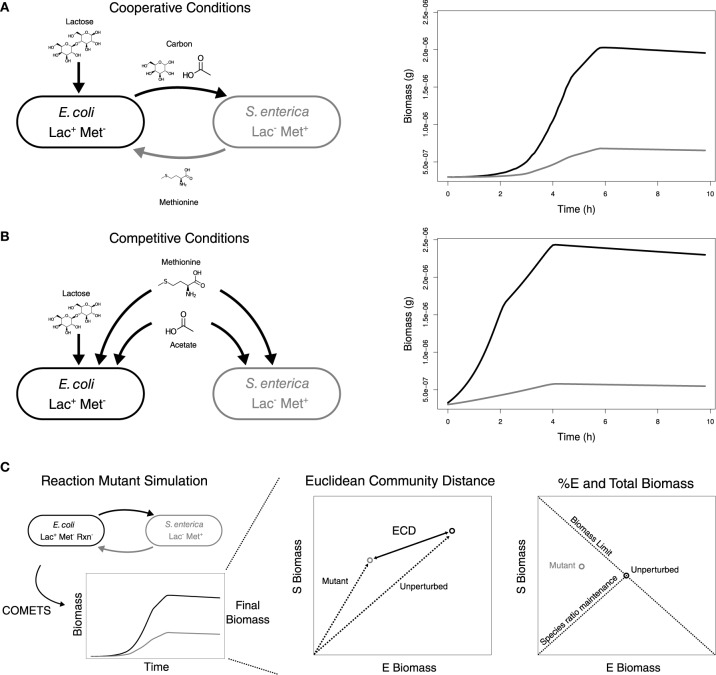
**Metabolic interactions in the unperturbed community and metrics for the analysis of the impact of perturbations**. Growth conditions and metabolic interactions between *E. coli* and *S. enterica* with growth data from COMETS for **(A)** Cooperation and **(B)** Competition. **(C)** Diagram of simulation data, ECD calculation, and %E and BM determination. Also, indicated are expected correlations for maintenance of species ratios and theoretical limits for BM.

Here we use metabolic modeling to determine the robustness of different types of pair-wise interactions. Specifically, we model interactions in a synthetic system involving *Escherichia coli* and *Salmonella enterica* that were previously engineered to depend on each other through the exchange of metabolites (Harcombe, 2010; Chubiz et al., 2014). In lactose minimal media, the species form an obligate mutualism as *S. enterica* relies on carbon byproducts from *E. coli* and *E. coli* in turn is dependent on methionine from *S. enterica* (Figure 1A). However, if acetate and methionine are added to the environment the species are no longer dependent on one another and instead compete for resources (Figure 1B). We investigated the impact of mutations (i.e., genetic perturbations) in each of these environments by systematically knocking out every reaction in the *E. coli* network one at a time and simulating community growth. We determined how each mutation influenced community properties, and compared the variance in properties when the species were competing or cooperating (Figure 1C). Based on the ecological principles discussed above, we expected that in the cooperative system species ratios would be robust to the effect of mutations, while in the competitive scenario total biomass would be robust. Finally, to investigate the generality of our findings we repeated the analysis by introducing mutations into the *S. enterica* model. The robustness of community phenotype to genetic perturbations has important implications for the dynamics of natural communities and informs design principles for engineered systems.

## Materials and methods

### Experimental system

We tested the genetic robustness of an engineered model system involving *E. coli* and *S. enterica* (Harcombe, 2010; Chubiz et al., 2014). In this system *E. coli* is unable to produce its own methionine because *metB* has been deleted from the genome. *S. enterica* excretes methionine as a result of experimental evolution (Harcombe, 2010). To simulate the dynamics of this system we used previously published genome-scale metabolic models of *E. coli* (iJO_1366, Orth et al., 2011), and *S. enterica* (iRR_1083, Raghunathan et al., 2009). The *E. coli* model was modified by blocking flux through the reaction that corresponds to *metB*, and the *S. enterica* model was modified to excrete methionine (Harcombe et al., 2014). Methionine excretion was encoded by coupling a secretion flux to the biomass production flux in *S. enterica*. This modification caused the model to excrete 0.5 mmol/gDW of new biomass (Harcombe et al., 2014). It has been shown that these modified models generate growth dynamics that quantitatively match laboratory observation (Harcombe et al., 2014). We refer to these initial modified models as unperturbed.

Libraries of genetically perturbed mutants were then computationally created for each species. A knockout mutant was created for each reaction in each species. Reactions were knocked out by setting the lower and upper bounds on the reaction in the model file to zero. This corresponds to 2583 mutants of *E. coli* and 1286 mutants of *S. enterica*. Note that this systematic blockage of individual reactions is slightly different than the approach of knocking out individual genes (Orth et al., 2011). Both knocking out individual reactions or all reactions associated with individual genes provide relevant information about the effect of genetic perturbations. We compared our results against previous computational experiments in which genes were removed and found that both displayed similar agreement with empirical observations. We discuss this comparison further in the results section.

### Computer simulations

The impact of each genetic perturbation was tested in a community context using the Computation of Microbial Ecosystems in Time and Space (COMETS) platform (Harcombe et al., 2014). Each perturbed mutant was paired with an unperturbed partner and the biomass of each species was assayed after 10 h of simulated growth. Simulations were initiated with 3 × 10^−7^ g biomass of each bacterial species. Growth was simulated in the minimal media used in previous work (Harcombe et al., 2014). The cooperative system was tested in an environment with 1.2 × 10^−5^ mmol lactose as the limiting reagent. The competitive environment was generated by adding unlimited acetate and methionine to the lactose minimal media. In this case the limiting reagent became the oxygen, which was set at 6.2 × 10^−5^ mmol in all scenarios. Each simulation was performed in a one by one grid with edges set to 0.5 mm. The time step was 0.1 h. The Michaelis-Menten uptake parameters were set to a default of *K_m_* = 10 μM, and *V_max_* = 10 mmol/g/h for all metabolites (Harcombe et al., 2014). The death rate was 0.01/h. COMETS simulations were performed on a 48-core computer with 64 GB of memory using COMETS version 1.3.3 (http://comets.bu.edu). Simulation data was parsed and tabulated with custom Python scripts (available on request).

### Data analysis

Three primary metrics were used to assess changes in community-level simulation outcomes. The first metric, which we term Euclidean community distance (ECD), was used to measure total community change. It is described by the following relationship:

$$ECD=100^{*}\sqrt{\frac{{\left(E-E_{Unperturbed}\right)}^{2}+{\left(S-S_{Unperturbed}\right)}^{2}}{E_{Unperturbed}^{2}+S_{Unperturbed}^{2}}}$$

where *E* is the simulated biomass of *E. coli* and *S* is the simulated biomass of *S. enterica* at the end of the simulation, i.e., 10 h after inoculation. Effectively, ECD is a percent deviation in biomass of all species relative to the observation in the absence of mutations (i.e., the deviation from our unperturbed community, Figure 1C). ECD = 0 for a genetically perturbed community in which both organisms reached the same biomasses observed in the unperturbed case.

While ECD gives an integrated view of the effects of mutations on community state, it does not give specific information on how the ratio of species (i.e., community composition) is influenced by genetic perturbation. To explore the robustness of community composition, we used the normalized percentage of *E. coli* in the community (%E), defined as:

$$\%E=\frac{E}{E+S}/\frac{E_{Unperturbed}}{E_{Unperturbed}+S_{Unperturbed}}$$

Finally, to investigate the robustness of community function we used a measure of community productivity. The normalized biomass (BM), was defined as.:

$$BM=\frac{E+S}{E_{Unperturbed}+S_{Unperturbed}}$$

As described in the results section, knockouts leading to changes in %E or BM values ≥1% of the unperturbed %E and BM were deemed to be substantive. The 1% cutoff was chosen because it eliminated small effects that arose from rounding or randomized calculation order in simulations.

To compare the robustness of cooperative and competitive communities for each of our metrics we used a mean-centered Levene test to evaluate the equality of variances in the face of mutation. If mutations in one context (i.e., cooperative or competitive) lead to a significantly smaller standard deviation in a community metric, that context was deemed more robust to genetic perturbation. The Levene test determines whether two groups have equal variance, and does not require the data to be normally distributed. Statistical significance was based on *P* < 0.05. The tests were implemented in R with the lawstat library. It should be noted that 2.2 × 10^−16^ is the lower limit of significance values in this package. Several of our tests returned values at this limit.

## Results

### Description of growth in the absence of genetic perturbation

The unperturbed *E. coli* and *S. enterica* followed similar growth trajectories in both cooperation and competition. *E. coli* was always the dominant community member, consistent with what was observed in prior simulations and experimental testing (Harcombe et al., 2014). In the lactose environment that required cooperation (Figure 1A) the community reached a total final biomass of 2.60 × 10^−6^ g, of which 75.1% was *E. coli*. Competition between species was created by providing excess acetate and methionine in the environment (Figure 1B). With the additional metabolites, the community reached a final biomass of 2.85 × 10^−6^ g, and the frequency of *E. coli* increased to 80.7%. The competitive scenario also allowed the bacteria to start growing sooner, as they could extract all necessary metabolites from the environment rather than waiting to obtain them from a partner.

### Validating the growth of genetically perturbed mutants

To determine the validity of our genetic perturbation models we first tested our ability to predict essential reactions in monoculture by comparing against previous work. Essential reactions in our simulations were defined as those whose removal allowed the mutant to grow less than 2% relative to the unperturbed organism, consistent with common empirical approaches (Baba et al., 2006). *E. coli* was found to have 287 essential reactions when simulated in monoculture with lactose, acetate and methionine.

To determine the validity of our genetic perturbation predictions, we compared them against previous experimental and computational analyses. Typically, perturbation studies investigate the loss of genes rather than the loss of specific reactions, as investigated in the current study. In order to compare our predictions against previous work, we mapped each reaction back to a gene. We compiled a list of 975 genes whose removal would lead to the loss of at least one metabolic reaction. Of this list, 198 genes were deemed essential based on the reactions that we found to be necessary for growth. We compared our predictions of gene essentiality against previous experimental assays of growth of *E. coli* knockout mutants in minimal media (summarized in Orth et al., 2011). We found that our predictions on essentiality of a gene matched empirical observations 90% of the time. Previous computational work—in which genes, rather than reactions, were knocked out—found a similar 91% agreement with experimental work (Orth et al., 2011).

### Genetic robustness of *E. coli* in different ecological contexts

We began by examining how ecological context influenced the robustness of a single organism. *E. coli* had fewer essential reactions when competing against *S. enterica* than when cooperating with it. When *E. coli* was competing, the same set of 287 reactions were found to be essential as when the bacteria was grown in monoculture; this seems reasonable given that the initial nutrients provided were the same. During cooperation, an additional 10 reactions were essential for growth. These reactions involved sugar transport (glucose and galactose exchange), glycolytic reactions (phosphoglycerate kinase, phosphoglycerate mutase, phosphoglyerate isomerase, and enolase), and maintenance energy. Metabolites must flow through these reactions in the initial stages of consortium growth, when *E. coli* is metabolizing carbon but not yet creating biomass. In essence the reactions help “jumpstart” the metabolic exchange between *E. coli* and *S. enterica*.

Mutations in non-essential reactions created greater variance in final *E. coli* biomass when the species were competing. Mutations in reactions that were essential in any context were excluded from the analysis. The variance between *E. coli* mutants was significantly bigger when the species were competing than when *E. coli* was cooperating with *S. enterica* (σ_Cooperation_ = 5.54 × 10^−8^ g, σ_Competition_ = 9.67 × 10^−8^ g, Levene Test *P* = 1.76 × 10^−15^).

### Cooperation minimizes change in the community

**Figure 2:**
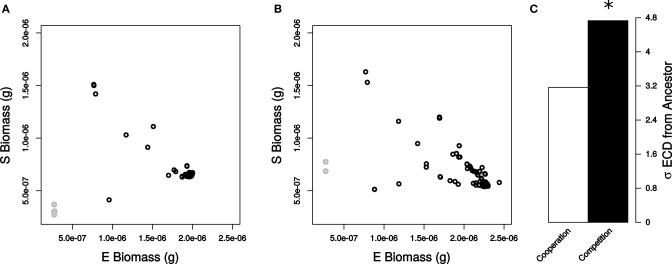
**Community state resulting from genetic perturbations in *E. coli***. Final biomass of each simulated species when bacteria were **(A)** cooperating and **(B)** competing. Non-essential reactions (black) and essential reactions (gray) are displayed in each plot. **(C)** Standard deviations of ECD values for all non-essential reactions for cooperation (white) and competition (black). Deviations in ECD were significant (star) by a Levene Test (*P* < 2.2 × 10^−16^).

After analyzing robustness of a single organism in different ecological contexts, we moved on to analyze the impact of mutations on community properties. Loss of function mutations in *E. coli* generated considerable variation in final *E. coli* and *S. enterica* biomass (Figures 2A,B). We quantified the amount by which each mutation altered the community by calculating a normalized Euclidean community distance (ECD, see Materials and Methods) when the species were competing or cooperating (Figures S1, S2). We first left out of the analysis simulations involving essential reactions to better quantify the impact of non-lethal mutations on altering *E. coli* and *S. enterica* biomass across each environment. Interestingly, we found that the cooperative relationship displayed significantly lower sensitivity to loss of function mutations as compared to competition (σ_ECD,Cooperation_ = 3.16 and σ_ECD,Competition_ = 4.73, Levene Test *P* < 2.2 × 10^−16^, Figure 2C). Upon including essential reactions into the analysis, we obtained similar, statistically significant trends (σ_ECD,Cooperation_ = 26.7 and σ_ECD,Competition_ = 27.1, Levene Test *P* < 2.2 × 10^−16^).

### Species ratios are more robust to genetic perturbations in the cooperative systems

**Figure 3:**
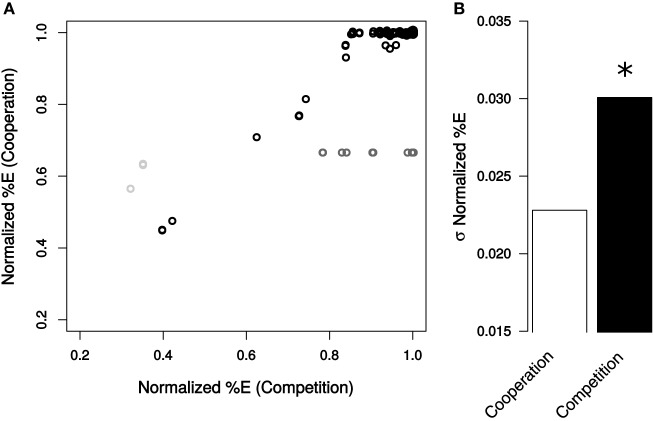
**Community composition resulting from genetic perturbations in *E. coli*. (A)** Normalized %E values caused by each mutation in cooperation and competition environments. Non-essential reactions are displayed in black, essential reactions in dark gray, and reactions essential for cooperation only in light gray. **(B)** Standard deviation of normalized %E for all non-essential reactions for cooperation (white) and competition (black). Deviations in normalized %E were determined to be significant (star) by a Levene Test (*P* = 6.47 × 10^−11^).

The percent of *E. coli* in the community was more resilient to mutation when the bacteria were cooperating (Figure 3). In this analysis, the final fraction (in %) of *E. coli* was standardized to those observed in the absence of perturbation in each growth condition. Mutations had a significantly smaller impact on the distribution of %E in the cooperative system than in the competitive scenario (σ_%E,Cooperation_ = 0.023, σ_%E,Competition_ = 0.031, Levene Test *P* = 6.47 × 10^−11^). This trend holds whether or not essential genes are included in the calculations (Levene Test *P* < 2.2 × 10^−16^).

Consistent with these observations on variance, the number of mutations found to alter the final composition of cooperative communities was also lower (Figure 3A). When *E. coli* was competing with *S. enterica*, 111 non-essential reactions altered species ratios by more than 1% when removed, while only 14 knockouts influenced the mutualism (Table S1). The reactions whose deletions cause the biggest effects tend to overlap between the two cases, and there is a correlation between the effects of the perturbations under each growth condition (*R*^2^ = 0.73). In both cases, loss of oxygen utilization in *E. coli* substantially shifted the community toward a greater percentage of *S. enterica*. Other reactions with large effects on community composition were ATP synthase, galactokinase and triose-phosphate isomerase.

### The total community biomass is not stabilized by competition

**Figure 4:**
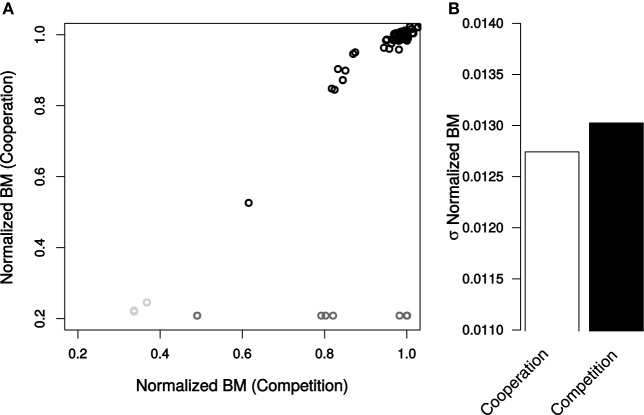
**Total community biomass resulting from genetic perturbations in *E. coli*. (A)** Normalized BM values caused by each mutation in cooperation and competition environments. Non-essential reactions (black), essential reactions (dark gray), and reactions essential for only cooperation (light gray). **(B)** Standard deviation of normalized BM for all non-essential reactions. Deviations in normalized BM were determined to be not significant by a Levene Test (*P* = 0.063).

In contrast to expectation, competition did not make biomass more resilient to genetic perturbations (Figure 4). The effects of mutations on community biomass were indistinguishable whether the bacteria were cooperating or competing (σ_BM,Cooperation_ = 0.0127, σ_BM,Competition_ = 0.0130, Levene Test *P* = 0.0634, Figure 4B). Interestingly, however, if essential reactions are taken into consideration, biomass is more stable in a competitive environment (σ_BM,Cooperation_ = 0.25, σ_BM,Competition_ = 0.21, Levene Test *P* = 1.88 × 10^−8^).

The number of mutations that altered biomass was higher under competition than under cooperation. In the competitive environment 104 mutations changed the total biomass by >1% relative to the unperturbed system (Table S1). When the two bacteria were cooperating, only 51 knockouts had an impact on final productivity. The effects of reaction removal on total biomass were highly correlated between the two scenarios (*R*^2^ = 0.79). In both cases the most significant outliers were ribose-5-phosphate isomerase, ATP synthase, and O_2_ exchange.

### In *S. enterica*, cooperation stabilizes species ratios but not total biomass

**Figure 5:**
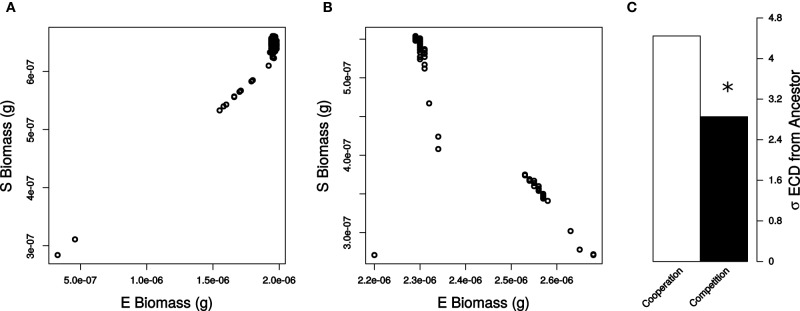
**Individual species biomass yields from COMETS simulations for (A) cooperation and (B) competition for all systematic reaction knockouts in *S. enterica* co-cultured with wild-type *E. coli***. Non-essential reactions (black) and essential reactions (gray) are displayed in each plot. **(C)** Standard deviations of ECD for non-essential reactions values for cooperation (white) and competition (black). Deviations in ECD were significant (star) by a Levene Test (*P* = 0.0062).

To test the generality of our findings, we also examined the impact of knocking out each reaction in the *S. enterica* model and again simulating growth of each mutant when competing and cooperating with *E. coli*. Mutations in *S. enterica* created very different distributions of final species densities than mutations in *E. coli* (Figure 5). Notably, *S. enterica* mutants more closely followed the expectation of cooperation maintaining the species ratios and competition maintaining total biomass.

Communities in which *S. enterica* mutants were competing with *E. coli* showed less change in our composite distance metric ECD, than did communities of mutualists (σ_ECD,Cooperation_ = 0.044, σ_ECD,Competition_ = 0.028, Levene Test *P* = 0.00622, Figure 5B). However, competition increased the variance in species ratios (σ_%E,Cooperation_ = 0.014, σ_%E,Competition_ = 0.019, Levene Test *P* = 0.00386).

Total biomass showed a different trend relative to what was observed when *E. coli* was perturbed. Loss of reactions in *S. enterica* created less biomass change in the competitive community than when the bacteria cooperated (σ_BM,Cooperation_ = 0.042, σ_BM,Competition_ = 0.007, Levene Test *P* = 0.00179). Mutations in *S. enterica* caused less variance in biomass during competition, and more variance in biomass during cooperation than mutations in *E. coli*. As a result, *S. enterica* followed the common assumption that competition should reduce the impact that perturbations have on community biomass.

## Discussion

There has been a great deal of work on the robustness of genomes and parallel work on the stability of communities. However, there are scant examples of studies that link these two approaches (Klitgord and Segrè, 2010). We investigated the link between community and genetic robustness by using a novel platform that predicts community dynamics from the behavior of genome-scale metabolic networks. We found that the robustness of the *E. coli* genome changed significantly in different ecological contexts. Perhaps more surprisingly, we found that cooperation stabilized the community against perturbations in *E. coli*. Cooperation reduced the variance in species ratios and lead to no more variance in total biomass relative to the competitive system. Interestingly, the opposite trends were observed on the community when perturbations were introduced to *S. enterica*. In this case, competition stabilized total biomass in the face of metabolic perturbations.

We found that mutations generated less variance in *E. coli* density when the bacterium was cooperating with *S. enterica* than when the two species were competing. This finding is consistent with previous work showing that genetic robustness of a genome can change in different environments (Baba et al., 2006). Furthermore, the direction of the change is reasonable. When *E. coli* is competing, mutations that slow its rate of growth should lead to a reduction in final yield, as *S. enterica* acquires more of the available resources. In contrast, when the species are cooperating *S. enterica* is less able to outpace its partner so *E. coli* mutants with slow growth can still reach a similar final density as the unperturbed strain even if it takes longer. Interestingly, despite the difference in robustness there was still a strong correlation between the effects of knockouts in each growth condition. The correlation was substantially strengthened by the large effect mutations. One of the perturbations that had the largest effect was forcing *E. coli* to ferment. While knocking out oxygen transport is clearly not feasible, it is possible to modulate *E. coli* fermentation through ArcAB and FNR (Compan and Touati, 1994; Becker et al., 1996).

Beyond a single organism we were interested in the robustness of communities to mutation. We found that cooperative communities were robust to genetic perturbations in *E. coli*. The community state, as measured by ECD, was changed less by mutations in the cooperative system than the competitive system. This finding is consistent with the observation by both Shou et al. and Harcombe et al. that synthetic mutualisms are highly robust to starting ratios and will converge to an equilibrium community composition even from divergent initial conditions (Shou et al., 2007; Harcombe et al., 2014). However, they are in contrast to the findings by Kim et al. that precisely defined spatial structure was necessary to stabilize the species ratios in a tri-partite mutualism (Kim et al., 2008). This discrepancy in results is largely based on whether a species is capable of outcompeting its symbiotic partners. In the case that we modeled, and the cross-feeding yeast strains of Shou et al. frequency dependent dynamics ensued from the fact that species' growth rates were limited by excretions from the most rare member of the community. In contrast, in the system of Kim et al. species were able to outcompete the partners on which they relied. This lack of frequency dependence may be more common as the number of partners increases (May, 1972). Consistent with this assertion, two recent studies using randomly generated matrices of species interactions found that networks of mutualists were less stable than networks of competitors (Allesina and Tang, 2012; Mougi and Kondoh, 2012). In future work it will be informative to investigate how more complex networks of species respond to genetic perturbations.

Competition is often assumed to stabilize community productivity (Gonzalez and Loreau, 2009), however this expectation was contradicted by our observation that mutations in *E. coli* produced equivalent changes in total biomass in the two ecological contexts. Compensatory dynamics are thought to arise in competitive communities because if the abundance of one species is reduced another species will simply increase thereby maintaining biomass (though see Loreau and de Mazancourt, 2013). Our system did not behave this way in part because there was a substantial difference in the efficiency of the two species. In the competition scenario, the two species were competing over oxygen. A cell engaged in fermentation of a sugar (i.e., *E. coli*) can make far more biomass per molecule of oxygen than a cell that is consuming acetate. By constraining the ratio of *E. coli* to *S. enterica* the cooperative interaction allowed more utilization of the limiting nutrient by the more productive species. In support of this assertion if mutations in essential reactions in *E. coli* are included in the analysis then the opposite trend is observed. If *E. coli* is unable to grow, then growth of *S. enterica* increases community productivity and stabilizes biomass as generally predicted. Furthermore, if mutations are put in the less efficient *S. enterica* partner then the compensatory effects of competition are again observed. These results highlight the utility of connecting metabolic mechanisms to the study of community dynamics. Genome-scale metabolic models make it possible to determine how community dynamics will be influenced by changes in the efficiency of resource utilization.

Perturbations to *S. enterica* had a significantly different effect than mutations to *E. coli*. Part of this effect may be an artifact of model size. The *S. enterica* model contains fewer reactions (1286 vs. 2583 in *E. coli*) and therefore a different subset of mutations is being tested. Specifically, the smaller network concentrates reactions that are more directly involved in central carbon metabolism. More interestingly, the specificity of exchanged metabolites influences the robustness of the interactions. *E. coli* obtains exclusively methionine from *S. enterica*, while *S. enterica* can grow on any number of different carbon excretions. The mutations with largest effect in *E. coli* were those that altered the currency of metabolic exchange, such as galactokinase, which forced the cells to excrete unreduced forms of carbon. Supplying different carbon sources to *S. enterica* broke the constraint on species ratios in cooperation, and improved the resources available to *S. enterica* in the competitive environment. In contrast, mutations in *S. enterica* did not change the nature of the metabolic interactions in meaningful ways in either environment (i.e., mutations did not change the identity of the compound *S. enterica* provided to *E. coli*). This again highlights the utility of connecting metabolic mechanisms to species interactions to understand community behavior.

Cooperation and conflict drive dynamics in natural and engineered communities. To predict and ideally control the dynamics of these communities it will be necessary to understand how they respond to genetic perturbations. Here we investigate the connection between ecological and genetic robustness. Going forward it will be interesting to determine how evolution acts on the variation that mutation can generate in a community context. This will involve looking at the relative fitness of each mutation rather than the effects of each mutation independently. Genome-scale metabolic models provide a powerful approach for mechanistically investigating the feedback between ecological and evolutionary processes, as well as connecting the activity of genes to the function of ecosystems.

## Author contributions

WH and LC designed the experiment, analyzed the data and wrote the manuscript. BG generated data, analyzed data and helped with writing. DS helped with data analysis and writing of the manuscript.

### Conflict of interest statement

The authors declare that the research was conducted in the absence of any commercial or financial relationships that could be construed as a potential conflict of interest.

## Supplementary material

Figure S1 **Histogram of ECD values for cooperation simulations**.

Figure S2 **Histogram of ECD values for competition simulations**.

Table S1 **Reactions identified as having >1% change in normalized %E or BM relative to the unperturbed state**.
